# Supplementary material for: Knowledge, practice and associated factors towards the prevention of COVID-19 among high-risk groups: A cross-sectional study in Addis Ababa, Ethiopia
Source: PLoS One. 2021 Mar 11;16(3):e0248420. doi: 10.1371/journal.pone.0248420 (PMC7951807; doi:10.1371/journal.pone.0248420)
Supplement: S1 File — (DOCX) [file pone.0248420.s002.docx]

|  | **Annex I: Data collection tool** | |  |
| --- | --- | --- | --- |
|  | **Question to assess the Community knowledge and practice towards the prevention of COVID19** | | |
|  | Redcap serial number_________________________ | |  |
|  | Name of data collector________________________ | |  |
|  | Data collector telephone_______________________ | |  |
|  | Form completion date_________________________ | |  |
|  | Full name sample collector____________________ | |  |
| 101 | Sex | Male…….….……….1 |  |
|  |  | Female………...…….2 |  |
| 102 | Age | ____________ |  |
| 103 | Respondent address Region/City administration | _____________________ |  |
| 104 | Nationality | _____________________ |  |
| 105 | Patient occupation (specify location/facility) | Health care worker…………..1 |  |
|  |  | Working with animals……….2 |  |
|  |  | Driver/assistant………….…..3 |  |
|  |  | guard kiosk……………….…4 |  |
|  |  | Grocery Student…………….5 |  |
|  |  | Other specify………………..6 |  |
| 106 | Religion | Orthodox……………………1 |  |
|  |  | Muslim……………………..2 |  |
|  |  | Protestant…………………..3 |  |
|  |  | Others…...…………………4 |  |
| 107 | What is your monthly income | less than 1000 birr…………1 |  |
|  |  | 1001-3000 birr…………….2 |  |
|  |  | 3001-10000 birr…………..3 |  |
|  |  | More than 10000………….4 |  |
| 108 | How does corona transmitted | Direct contact with Corona patient……..A |  |
|  |  | During breathing…………………….....B |  |
|  |  | Cough and sneeze……………………...C |  |
|  |  | Eating and drinking contaminated food with corona………………………………….D |  |
|  |  | Other…………………………………...E |  |
| 109 | What is prevention mechanism of corona | Social distancing ………………………A | |
|  |  | Hand washing………………………….B |  |
|  |  | Use of facemask……………………….C |  |
|  |  | Isolation/quarantine other……………..D |  |
| 110 | Which method are you practicing to prevent corona | Social distancing………………..……..A |  |
|  |  | Hand washing………………...……….B |  |
|  |  | Using facemask …..…………………..C |  |
|  |  | Stay home…….…..…………………..D |  |
|  |  | Other……………..…………………..E |  |
| 111 | Average daily water intake in the last five days in Letter | _____________ |  |
| 112 | How frequent do you take water per day for the last 5 day in number | ______________ |  |
|  | Part II: Symptom, Complication and Preexisting Conditions | |  |
| 201 | History of fever Yes | Yes………1 |  |
|  |  | No………2 |  |
| 202 | Did you visited health facility visit for the fever | Yes………1 |  |
|  |  | No………2 |  |
| 203 | Maximum temperature recorded in °C | ________ |  |
| 204 | Have you ever Respiraatory sypmtom | Yes………1 | If the Ans is 2 skip to Q207 |
|  |  | No………2 |  |
| 205 | Type of respiratory symptoms | Cough……………..………A |  |
|  |  | Runny nose……….……….B |  |
|  |  | Sore throat…………………C |  |
|  |  | Shortness of breath……...…D |  |
|  |  | Other symptoms….………..E | |
| 206 | Have other symptoms otherthan respiratory | Yes………1 |  |
|  |  | No………2 |  |
| 207 | Other experianced symptoms Chills Vomiting Nausea Diarrhoea Headache Rash Conjunctivitis Muscle aches Joint ache | Chillis……………………….A |  |
|  |  | Vomiting……………………B |  |
|  |  | Nausea……………………...C |  |
|  |  | Diarrhoea…………………...D |  |
|  |  | Headache………...…………E |  |
|  |  | Rash………………………..F |  |
|  |  | Conjuctivites……………….G |  |
|  |  | Muscle aches………………H |  |
|  |  | Joint ache…………………..I |  |
|  |  | Loss of appetite……………J |  |
|  |  | Nose bleeding…………….K |  |
|  |  | Fatigue……………………L |  |
|  |  | Seizures…………………..M |  |
|  |  | Altered consciousness…….N |  |
|  |  | Other neurological signs…..O |  |
|  |  | Others please specify………P |  |
| 208 | Have you ever had hospitalization with in 15 days ago | Yes…………………1 | If the Ans is 2 skip to Q210 |
|  |  | No………………….2 |  |
| 209 | Date of first hospitalization | ____________________ |  |
| 210 | Patient pre-existing condition(s) | Pregnancy………………………………A |  |
|  |  | Obesity…………………………………..B |  |
|  |  | Cancer Diabetes…………………………C |  |
|  |  | HIV/other immune deficiency…………..D |  |
|  |  | Heart disease…………………………….E |  |
|  |  | Asthma (requiring medication)………….F |  |
|  |  | Chronic lung disease (non-asthma)…….G |  |
|  |  | Chronic liver disease…………………..H |  |
|  |  | Chronic hematological disorder………..I |  |
|  |  | Chronic kidney disease………………..K |  |
|  |  | Chronic neurological impairment/disease...L |  |
|  |  | Organ or bone marrow recipient……….M |  |
|  |  | Other pre-existing condition(s)………..N |  |
| 212 | Is the participant pregnant? Yes | Yes…………………………..1 | If the Ans is 2 or 3 skip to Q301 |
|  |  | No…………………………...2 |  |
|  | No Unknown | Not applicable……………….3 |  |
| 213 | Period of Pregenancy | Fisrt trimester…………………1 |  |
|  |  | Second trimetster……………..2 |  |
|  |  | Thired trimester……………….3 |  |
|  | **Part III: Healthcare Interactions and Exposure Before Symptom** | |  |
| 301 | Have you ever contacted 8335 Yes | Yes…………………….1 |  |
|  |  | No……………………..2 |  |
| 302 | Have you evere visited health facility | Yes…………………….1 | If the ans 2 skip to Q 304 |
|  |  | No……………………..2 |  |
| 303 | Have you visited emergency department Yes No Unknown | Yes…………………….1 |  |
|  |  | No……………………..2 |  |
| 304 | Have you travelled within the last 14 days domestically? No | Yes…………………….1 | If the ans 2 skip to Q 308 |
|  |  | No……………………..2 |  |
| 305 | Date of travel(dd/mm/yyyy) | _________________ |  |
| 306 | Regions visited: | _________________ |  |
| 307 | Cities visited: | ________________ |  |
| 308 | Have you travelled within the last 14 days internationally? No | Yes…………………….1 | If the ans 2 skip to Q 312 |
|  |  | No……………………..2 |  |
| 309 | Dates of travel | _______________ |  |
| 310 | Countries visited: | ______________ |  |
| 311 | Cities visited: | _____________ |  |
| 312 | In the past 14 days, have you had contact with anyone with suspected/confirmed COVID-19? No | Yes…………………….1 |  |
|  |  | No……………………..2 |  |
| 313 | Have you attended festival or mass gathering in the past 14 days ? No | Yes…………………….1 |  |
|  |  | No……………………..2 |  |
| 314 | Have you visited outpatient treatment facility in the past 14 days No | Yes…………………….1 |  |
|  |  | No……………………..2 |  |
| 315 | Have you visited traditional healer in the past 14 days? No | Yes…………………….1 |  |
|  |  | No……………………..2 |  |
| 316 | Have you been hospitalized in the past 14 days for any reason not COVID No | Yes…………………….1 |  |
|  |  | No……………………..2 |  |

**አማረኛ ቃለ መጠየቅ**

| ለኮሮና ምርመራ ለመጡ ሰዎች ካሁን በፊት ስለበሽታው መተላለፊያና መከላከያ ያላቸውን እውቀት እንዲሁም በሽታውን ለመከላከል ስላደርጉተ መከላከያ ዘዶዎች የሚጠየቅ ቃለ መጠየቅ | | | |
| --- | --- | --- | --- |
|  | የተመርማሪው መላያ ቁጥር | ________________________________________ |  |
|  | መርጃውን የሰበሰበው ባለሙያ | ________________________________________ |  |
|  | መርጃውን የሰብሳቢው ስልክ ቁጠር | ________________________________________ |  |
|  | ፎርሙ የተሞላበት ቀን | ________________________________________ |  |
|  | የመርማሪው ስም | _______________________________________ |  |
| **ጥ.ቁ** | **ጥያቄ** | **ምርጫ** | **ቀጣይ** |
| 101 | ፆታ | ወንድ……………..1 |  |
|  |  | ሴት …………………..2 |  |
| 102 | እድሜ በሙሉ አመት |  |  |
| 103 | የመኖሪያ ቦታ |  |  |
| 104 | ዜግነት |  |  |
| 105 | የተመርማሪው የስራ አይንት | ጤና ባለሙያ …………..1 |  |
|  |  | እንስራት አረቢ …………2 |  |
|  |  | ሹፌረ…………………….3 |  |
|  |  | የጥበቃ ስራ………………..4 |  |
|  |  | ስቅ ላይ የሚስራ………..5 |  |
|  |  | ተማረ ……………….6 |  |
|  |  | ሌላ (ይጻፍ) ……………..7 |  |
| 106 | ሄይማኖት | ኦረቶዶክስ ክረስቲያን…………………………………….1 |  |
|  |  | ሙስሊም………………………………………………….2 |  |
|  |  | ፕሮቴስታነት………………………………………………3 |  |
|  |  | ሌላ………………………………………………………….4 |  |
| 107 | የወር የገቢ መጠን ( በብር) | ከ 1000 በታች………………………………………….1 |  |
|  |  | ከ 1001 -3000…………………………………………2 |  |
|  |  | 3001-10000…………………………………………..3 |  |
|  |  | ከ10000 በላይ………………………………………….4 |  |
| 108 | ኮሮና በምን እና እንዴት እንደሚተላለፍ ቢንግሩኝ | በኮሮና ከተያዘ ሰው ጋር ቀጥታ በሆን ንክኪ……ሀ |  |
|  |  | በትንፋሽ…………………………………….ለ |  |
|  |  | በሳል ወይም በንትሻ ጊዜ……………...………ሐ |  |
|  |  | በኮሮና የተበከላ ምግብ እና ዉሀ በመውሰድ…..መ |  |
|  |  | ሌላ…………………………………………ሠ |  |
| 109 | ኮሮናን ለመከላከል ምን ማድርግ እንዳለብዎት ይንገሩኝ | አካላዊ እርቀትን በመጠበቅ……………..…….ሀ |  |
|  |  | እጅን በሳሙና በመታጠብ ዎይም ሳኒታይዘር በማጽዳት……………………………………ለ |  |
|  |  | የአፍ መሸፈኛ ጨንብል በመጠቀም………………………………….ሐ |  |
|  |  | በበሽታው ከተያዘን እራሳችንን በማግለል……………………….………….መ |  |
|  |  | ሌላ……………..……………………….…ሠ |  |
| 110 | የኮሮናነሰ በሽታ ለመከለለል ምን አይንት የመቀላቀያ መንገዶችን ትጠቀማለህ/ሽ | አካላዊ እርቀትን በመጠበቅ……………….………….ሀ |  |
|  |  | እጅን በሳሙና በመታጠብ ዎይም ሳኒታይዘር በማጽዳት……………………………………………………………ለ |  |
|  |  | የአፍ መሸፈኛ ጨንብል በመጠቀም………………………………………….ሐ |  |
|  |  | በቤት በመቆየት……………………..……………………መ |  |
|  |  | ሌላ………………………….…………………….ሠ |  |
| 111 |  |  |  |
| 112 | ላለፉት አምስት ቀናት በቀን ለምን ያህል ጊዜ ውሀ ተጠቀምክ በቁጥር | ____________________________ |  |
| 201 | በባለፉት ሁለት ሳምንት ትኩሳት ነበረብህ/ሽ | አዎ………………………………………………..1 |  |
|  |  | የለም…………………………………………….2 |  |
| 202 | ትኩሳት በነበርብዎት ስዓት ጤና ተቃም ሂደው ነበር | አዎ………………………………………………..1 |  |
|  |  | የለም…………………………………………….2 |  |
| 203 | አሁን ያለዎት የሙቀት መጠን | …………...በድግሪሴንቲግሬድ |  |
| 204 | የአተነፋፈስ ህመም ንሮብዎት ነበር | አዎ………………………………………………..1 | መልሱ 2 ከሆን ወደ ጥ.ቁ 207 |
|  |  | የለም…………………………………………….2 |  |
| 205 | ምን አይነት የአተነፋፈስ ህመም ነበር | የጉሮሮ መከርከር ወይም ከአፍጫ የሚዎጣ ፈሳሥ…………………………………………………………..ሀ |  |
|  |  | ሳል…………………………………………………………………ለ |  |
|  |  | የትንፋሽ ማጠር……………………………………………….ሐ |  |
|  |  | ሌላ (ይገለፅ)…………………………………….…...መ |  |
| 206 | ከአተንፋፈስ ዉጭ የታዩ ሌላ ምልክቶች | ብርድ ብረድ ማለት………...…………………….ሀ |  |
|  |  | ማስታወክ……………………………………....ለ |  |
|  |  | ተቅማጥ………………………………………..ሐ |  |
|  |  | ራስ ምታት……………………………………..መ |  |
|  |  | የሰውነት መንደብደብ ሽፍታ……………………..ሠ |  |
|  |  | የአይን መዥት ህመም………………………….ረ |  |
|  |  | ቁርትማት……………………………...……...ሰ |  |
|  |  | የመገጣጠሚያ ህመም……………………...…ሸ |  |
|  |  | የምግብ ፍላጎት ማጣት………………………..ቀ |  |
|  |  | የአፍንጫ መድማት…………………………...በ |  |
|  |  | ድካም………………………………………..ተ |  |
|  |  | ማንቀጥቀጥ…………………………………..ቸ |  |
|  |  | ያለመረጋጋት……………………………...….ኘ |  |
|  |  | ከአምዕሮ ጋር የተያያዘ የህምም ምልክቶት…….አ |  |
|  |  | ሌላ (ይገለፅ)………………………………..ከ |  |
| 207 | በዚህ 15 ቀን ውስት ታመው ሆስፒታል ገብተው ነበር | አዎ………………………………………………..1 | መልሱ 2 ከሆን ወደ ጥ.ቁ 209 |
|  |  | የለም…………………………………………….2 |  |
| 208 | ታመው ሆስፒታል የገቡበት ቀን መቼ ነበር |  |  |
| 209 | ተመርማሪው ያሉበት ተጎዳኝ ሁኔታዎት | ነፍሰጡረ……….………………………………….ሀ |  |
|  |  | ውፍርት……….…………………………………..ለ |  |
|  |  | የካንሰር በሽታ……….………………………….…ሐ |  |
|  |  | የስኮር በሽታ……….………………………………መ |  |
|  |  | ኤችአይቪ……….………………………………….ሠ |  |
|  |  | የልብ በሽታ……….…………………………………ረ |  |
|  |  | አስም……….……………………………………...ሰ |  |
|  |  | ብዙ ጊዝ የቆየ የሳንባ በሽታ…..………………………ቀ |  |
|  |  | ብዙ ጊዝ የቆየ የጉበት በሽታ……….…………………በ |  |
|  |  | ብዙ ጊዝ የቆየ ደም በሽታ……….……………………ተ |  |
|  |  | ብዙ ጊዝ የቆየ አዕምሮ ወይም የነርብ የበሽታ……….…ቸ |  |
|  |  | የ አጥንት የመጎልበት ችግረ……….………………….ኘ |  |
|  |  | ሌላ( ይጻፍ)……………..……….………………….አ |  |
| 210 | ተመርማሪወ/ታማሚው ነፍሰጡር ነው | አዎ……………………………………………..…………..1 | መልሱ 1 ወይም 2 ከሆን ወደ ጥ.ቁ 301 |
|  |  | የለም……………………………………...……………….2 |  |
|  |  | መጠይቁ አይሆናቸውም………………………….3 |  |
| 211 | የአግዝናው የጊዜ መጠን | የመጀመያው ሶስት ወር…………………………..1 |  |
|  |  | ስድስት ወር የሆነው………………………………2 |  |
|  |  | ከሰባት ወር በላይ የሆነው…………………………3 |  |
|  | Healthcare Interactions and Exposure Before Symptom | |  |
| 301 | በ8335 በነጻ የስልከ መስምረ ስለ ኮሮና መርጃ አግኝተው ነበረ | አዎ……………………………………………..…………..1 |  |
|  |  | አላገኘውም………………………………………2 |  |
| 302 | ባለፉት 14 ቀን ውስጥ ጤና ተቃም ሂደው/ጎብኝተው ነበረ | አዎ……………………………………………..…………..1 | መልሱ 2 ከሆን ወደ ጥ.ቁ 304 |
|  |  | የለም……………………………………...……………….2 |  |
| 303 | የጤና ተቃሙን ድንገተኛ ክፍል ሁደው/ጎብኝተው ነበረ | አዎ……………………………………………..…………..1 |  |
|  |  | የለም……………………………………...……………….2 |  |
| 304 | ባለፉት 14 ቀናት ውስጥ የአገር ውስጥ ጉዞ አድረግው ነበረ | አዎ……………………………………………..…………..1 | መልሱ 2 ከሆን ወደ ጥ.ቁ 308 |
|  |  | የለም……………………………………...……………….2 |  |
| 305 | የሄዱበት ቀን መቼ ነበረ |  |  |
| 306 | የሄዱበት ክልል |  |  |
| 307 | የሄዱበት ከተማ |  |  |
| 308 | ባለፉት 14 ቀናት ውስጥ ከአገር ውጭ ጉዞ አድረግው ነበረ | አዎ……………………………………………..…………..1 | መልሱ 2 ከሆን ወደ ጥ.ቁ 312 |
|  |  | የለም……………………………………...……………….2 |  |
| 309 | የሄዱበት ቀን መቼ ነበረ | __________________________ |  |
| 310 | የሄዱበት አገር | _________________________ |  |
| 311 | የሄዱበት ከተማ | _______________________ |  |
| 312 | ባለፉት 14 ቀናት ውስጥ በኮሮና ከተያዘ ሰው ጋር ግንኙነት ነበረዎት | አዎ……………………………………………..…………..1 |  |
|  |  | የለም……………………………………...……………….2 |  |
|  |  | እርግጠኛ አይደለውም……………………………….3 |  |
| 313 | ባለፉት 14 ቀናት ውስጥ ብዙ ህዝብ የተሰበሰበበት ፌስቲቫል ተሳትፍው/ ታድመው ነበር | አዎ……………………………………………..…………..1 |  |
|  |  | የለም……………………………………...……………….2 |  |
| 314 | ባለፉት 14 ቀናት ውስጥ ለተመላላሽ ህክምና ጤና ተቃም ሂደው /ጎብኝተው ነበረ | አዎ……………………………………………..…………..1 |  |
|  |  | የለም……………………………………...……………….2 |  |
| 315 | ባለፉት 14 ቀናት ውስጥ የባህል ህክምና ለማግኘት ሂደው ነበር | አዎ……………………………………………..…………..1 |  |
|  |  | የለም……………………………………...……………….2 |  |
| 316 | ባለፉት 14 ቀናት ውስጥ በጠና ታመው ሆስፒታል ተኝተው ነበር | አዎ……………………………………………..…………..1 |  |
|  |  | የለም……………………………………...……………….2 |  |
